# A Calcium-Deficient Diet in Rat Dams during Gestation and Nursing Affects Hepatic *11β-hydroxysteroid dehydrogenase-1* Expression in the Offspring

**DOI:** 10.1371/journal.pone.0084125

**Published:** 2014-01-10

**Authors:** Junji Takaya, Sohsaku Yamanouchi, Kazunari Kaneko

**Affiliations:** Department of Pediatrics, Kansai Medical University, Moriguchi, Osaka, Japan; The University of Manchester, United Kingdom

## Abstract

**Background:**

Prenatal malnutrition can affect the phenotype of offspring by changing epigenetic regulation of specific genes. Several lines of evidence demonstrate that calcium (Ca) plays an important role in the pathogenesis of insulin resistance syndrome. We hypothesized that pregnant female rats fed a Ca-deficient diet would have offspring with altered hepatic glucocorticoid-related gene expression and that lactation would modify these alterations.

**Methodology:**

We determined the effects of Ca deficiency during pregnancy and/or lactation on hepatic *11β-hydroxysteroid dehydrogenase-1* (*Hsd11b1*) expression in offspring. Female Wistar rats consumed either a Ca-deficient (D: 0.008% Ca) or control (C: 0.90% Ca) diet *ad libitum* from 3 weeks preconception to 21 days postparturition. On postnatal day 1, pups were cross-fostered to the same or opposite dams and divided into the following four groups: CC, DD, CD, and DC (first letter: original mother's diet; second letter: nursing mother's diet). All offspring were fed a control diet beginning at weaning (day 21) and were killed on day 200±7. Serum insulin and adipokines in offspring were measured using ELISA kits.

**Principal Findings:**

In males, mean levels of insulin, glucose, and Homeostasis Model Assessment of Insulin Resistance (HOMA-IR) were higher in the DD and DC groups than in the CC group. We found no difference in HOMA-IR between the CC and CD groups in either males or females. Expression of *Hsd11b1* was lower in male DD rats than in CC rats. *Hsd11b1* expression in male offspring nursed by cross-fostered dams was higher than that in those nursed by dams fed the same diet; CC vs. CD and DD vs. DC. In females, *Hsd11b1* expression in DC rats was higher than that in CC rats.

**Conclusions:**

These findings indicated that maternal Ca restriction during pregnancy and/or lactation alters postnatal growth, *Hsd11b1* expression, and insulin resistance in a sex-specific manner.

## Introduction

Calcium (Ca) plays an important role in the pathogenesis of insulin resistance syndrome and obesity, suggesting that hypocalcemia is a risk factor for developing these conditions [1], [2]. Several reports have shown an inverse relationship between Ca intake and body fat mass [2], [3], [4], [5]. The Coronary Artery Risk Development in Young Adults Study suggests an intriguing and exciting negative relationship between Ca intake or dairy consumption and obesity and insulin resistance syndrome in young adults [6]. Maternal malnutrition and the resulting low birth weight predispose offspring to various diseases, including adult-onset insulin resistance syndrome [7], [8], [9], [10]. Some experimental and observational findings from humans and animals indicate an association between maternal Ca intake during pregnancy and blood pressure in offspring [11], [12], but others do not [13]. Morley *et al*. reported that children whose mothers took Ca supplements during pregnancy had a better lipid profile at 9 years of age compared to children whose mothers did not take Ca supplements [14].

Glucocorticoids such as cortisol are important mediators in the regulation of cardiovascular and metabolic functions. Through activation of glucocorticoid or mineralocorticoid receptors, glucocorticoids impact vascular, adipose, liver, and kidney functions [15], [16]. 11β-hydroxysteroid dehydrogenase-1 (11β-HSD1, *Hsd11b1*) affects local cortisol production [17], [18]. 11β-HSD1 preferentially catalyzes the conversion of inactive 11-keto-glucocorticoids (cortisone in humans and 11-dehydrocorticosterone in rodents) into active 11β-hydroxyglucocorticoids (cortisol in humans and corticosterone in rodents), and by this mechanism, 11β-HSD1 modulates cell-specific glucocorticoid action [19], [20]. 11β-HSD1 activity has been shown to be elevated in obese humans with type 2 diabetes mellitus and metabolic syndrome [21], [22].

We previously reported that a low Ca diet alters glucocorticoid metabolism, leading to hepatic up-regulation of *Hsd11b1* in rats [23]. We also reported that feeding a Ca-restricted diet to pregnant rats results in hypomethylation and decreased expression from the *Hsd11b1* promoter in the liver of offspring at day 21 [24]. These findings show that maternal Ca deficiency during pregnancy can affect regulation of non-imprinted genes by altering epigenetic regulation of gene expression, thereby inducing different metabolic phenotypes. The epigenome is an important target of environmental modification. Dietary modification can have a profound effect on DNA methylation and genomic imprinting [25], [26]. A remarkable example of an environmental effect on the epigenome is the modification of glucocorticoid receptor methylation seen in the hippocampus of rat pups in response to maternal grooming [27]. Recently Yuen et al. reported that most fetal tissue-specific differentially methylated regions seem to reflect transient DNA methylation changes during development rather than permanent epigenetic signatures [28].

In this context, Ca can be seen as a strong candidate for a modulator of programming effects. We hypothesized that rat dams fed a diet lacking in Ca would produce offspring with altered expression of genes involved in hepatic glucocorticoid metabolism and that lactation would modify these alterations. The early nurturing environment has persistent influences on developmental programming of inter-individual differences in metabolic and endocrine function. Thus, the purpose of this study was to identify the effect of lactation by dams fed different Ca-containing diets on offspring, which were already programmed prenatally [29].

Regulation of hepatic 11β-HSD1 by the fetus or during lactation and its contribution to insulin resistance in the offspring are currently unknown. In this study, we fed pregnant rats a Ca-deficient diet and investigated the effect on insulin-related parameters and mRNA expression of *Hsd11b1* in the liver of offspring. In addition, we used a cross-fostering strategy to assess whether postnatal lactation by Ca-deficient dams contributed to the development of insulin resistance.

## Materials and Methods

### Animal procedures

Twelve-week-old female Wistar rats obtained from Shimizu Laboratories (Kyoto, Japan) were used. All rats were maintained on a 12-hour light/12-hour dark cycle. Virgin Wistar rats were divided into two dietary groups of five rats each and fed either a control diet (C: 0.90% Ca) or a Ca-deficient diet (D: 0.008% Ca) *ad libitum* for 3 wk. Experimental diets, the compositions of which are presented in Table 1, were prepared weekly in the laboratory. Rats were fed loose pellets in small metal dishes and were given free access to water. After 3 wk, the rats were mated with males who were fed a normal, control diet. The pregnant rats continued consuming their respective diets throughout the gestation and lactation periods.

**Table 1:** Ingredient composition of each diet fed to rats.

Ingredients	Control (g/100g diet)	Calcium deficient (g/100g diet)
Milk casein	24.50	24.50
Corn starch	45.50	45.50
Granulated sugar	10.00	10.00
Corn oil	6.00	6.00
Cellulose powder	5.00	5.00
α-Starch	1.00	1.00
Vitamin mix^1)^	1.00	1.00
Mineral mix^2)^	7.00	7.00
(Calcium)	(0.90)	(0.008)
Total	100.00	100.00

1), 2) refer Table S1 and Table S2.

In all litters, both to obviate the reported effects of litter size on metabolic function and to parallel commonly adopted protocols in the developmental programming literature, larger litters were reduced to six pups at 24 hours postpartum, maintaining equal sex ratios when possible. The fostering was done within 24 hours of birth (usually within 12 hours), and the animals were then disturbed as little as possible for the first 4–5 days after fostering. The following four groups were then established: CC (n = 18), in which offspring of control-diet dams were nursed by control dams (n = 3); DD (n = 18), in which offspring of Ca-deficient-diet dams were nursed by Ca-deficient-diet dams (n = 3); CD (n = 12), in which offspring of control-diet dams were nursed by Ca-deficient-diet dams (n = 2); and DC (n = 12), in which offspring of Ca-deficient-diet dams were nursed by control dams (n = 2). No pups remained with their original birth mother; the pups in all groups came from different litters. No pups were lost subsequent to cross-fostering. On day 21, six female and six male offspring were selected from the CC and DD groups by random removal. Six male and six female pups from each nutritional group were used for the study. The remaining 12 pups were not used. All offspring were weaned at 21 days onto on a control diet (0.90% Ca, supplied by CLEA Japan, Inc., Tokyo, Japan) and maintained on this diet until day 200±7. All 48 pups survived until day 200±7. Blood pressure and heart rate of conscious rats were measured with the tail-cuff method using a blood pressure monitor (Model MK-2000, Muromachi Kikai Co., Ltd., Tokyo, Japan) on the day before sacrifice. Animals were warmed to 35°C, and the mean of at least 10 measurements was taken. Body weight of offspring was measured every week from day 21 to day 200. No measurement of pup weight was made at birth because we did not wish to compromise the success of the cross-fostering by disturbing the pups. After fasting for 12 hours, the rats were decapitated with a guillotine, and blood samples were taken from the trunk. Livers were excised immediately, frozen in liquid nitrogen, and stored at −80°C. This study was carried out in strict accordance with the recommendations in the Guide for the Care and Use of Laboratory Animals of the National Institutes of Health. The protocol was approved by the Committee on the Ethics of Animal Experiments of Kansai Medical University (Permit Number: 11-012). All efforts were made to minimize suffering.

### Analysis of *Hsd11b1* and *cyclophilin* (*Ppia*) mRNA expression

Expression of mRNA in six offspring of each group was measured using real-time PCR. Total RNA was isolated from hepatic tissue samples using an RNeasy Mini Kit (QIAGEN Science, Germantown, MD) according to the manufacturer's instructions and quantified spectrophotometrically at an optical density of 260 nm. Details regarding the oligonucleotide primers for *Hsd11b1* and *Ppia* are listed in Table 2. Real-time PCR amplification of mRNA was performed in a total volume of 25 µl with a One Step SYBR PrimeScript RT-PCR Kit II (Takara Bio Inc., Tokyo, Japan) according to the manufacturer's recommendations. The amplification of mRNA was quantified using relative standard curve methods. To confirm the amplification of specific transcripts, melting curve profiles were produced at the end of each run. Samples were analyzed in duplicate, and expression of the individual transcripts was normalized [30] to expression of *Ppia* (QuantiTect assay QT00177394) [31], which did not differ among groups of offspring.

**Table 2:** Primer sequences used to amplify mRNA with real-time RT-PCR.

Gene		Primer(5′-3′)
*Hsd11b1*	Forward	TCATAGACACAGAAACAGCTTTGAAA
	Reverse	CTCCAGGGCGCATTCCT
*Ppia*	Forward	TTGGGTCGCGTCTGCTTCGA
	Reverse	GCCAGGACCTGTATGCTTCA

*Hsd11b1*, 11β-hydroxysteroid dehydrogenase 1; *Ppia*, Cyclophilin

### Serum measurements

#### Serum 11β-HSD1 and corticosterone levels

Whole blood from fasting rats was collected from the offspring of the four groups (n =  six males and six females in each group). Serum was separated by centrifugation. Serum 11β-HSD1 levels were detected using the Rat Corticosteroid 11-beta-dehydrogenase isozyme 1(HSD11B1) ELISA kit (CUSABIO, Wuhan, China). The lower limit of detection was 3.12 pg/ml. Serum corticosterone levels were detected using the Rat Corticosterone Enzyme Immunoassay Kit (Yanaihara Inc., Shizuoka, Japan). The lower limit of detection was 0.21 ng/ml.

#### Serum adipokine levels

Concentrations of leptin, insulin, and adiponectin were determined in serum from fasting rats using rat-specific ELISA kits (Morinaga, Yokohama, Japan (leptin and insulin); Otsuka Pharmaceutical, Tokyo, Japan (adiponectin)). The lower limit of detection was 0.4 ng/ml for leptin, 0.1 ng/ml for insulin, and 0.25 ng/ml for adiponectin. Glucose concentrations were measured using a glucose oxidase system (Arkray, Kyoto, Japan). Homeostasis Model Assessment of Insulin Resistance (HOMA-IR) is the product of fasting plasma glucose (mM) and the insulin concentration (ng/mL). Because no rat international unit has been established, the insulin units were converted to the human type for convenience. The equation used for this calculation is: 1 mg = 23.1 IU.

HOMA-IR  =  [Glucose] × [Insulin]/22.5 and was used as a measure of insulin resistance [32]. HOMA-β  = 20× [Insulin]/([Glucose] - 3.5)% and was used as a measure of β-cell function [33].

#### Serum ionized calcium levels

Serum ionized calcium levels were measured with an automatic ion selective electrode analyzer, Stat Profile pHOx Ultra (NOVA, Newton, MA), which provides results for ionized calcium in a measurement range of 0.10-2.70 mmol/l. The normalized ionized Ca represents the ionized Ca concentration at pH 7.40. The equation used for this calculation is as follows:
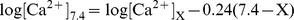
where X =  measured pH of the sample

[Ca^2+^]_X_ =  ionized Ca concentration in the sample at the measured pH

[Ca^2+^]_7.4_ =  normalized concentration of ionized Ca at pH 7.40

### Statistical analyses

Statistical analyses were performed using JMP 6 software (SAS Institute Inc., Cary, NC). Results are expressed as the means ± standard error (SE). Statistically significant differences among the groups were determined with two-way analysis of variance (ANOVA) with diet and sex as the main factors. If interactions were found, the data were split, and a one-way ANOVA was performed. A standard power calculation was performed on the results using JMP 6 software. Acceptable study power was agreed a priori to be ≥80% (type-I error of ≤0.20). P<0.05 was considered statistically significant.

## Results

Dams: No significant differences in body weight gain were found between Wistar female rats in the Ca-deficiency (n = 5) group and the control group (n = 5) at the mating. However, ionized Ca concentrations in plasma samples were lower (P<0.05) in the Ca-deficient group (1.31±0.05 mmol/l, n = 5) than in the controls (1.39±0.05 mmol/l, n = 5).

Pups: The litter size of six pups was uniform from postnatal day 1 until postnatal day 21. No significant differences in body weight were found at day 21 among the groups. The body weight of female DC offspring was higher than that in CC offspring at day 200±7 (P<0.05, Table 3). The body weight of male DC offspring was significantly higher than that in the other groups both on day 100 (P<0.05) and on day 200 (P<0.01, Table 4). No significant differences were observed among the groups for heart rate or systolic blood pressure.

**Table 3:** Profile of female offspring on day 200.

	CC	CD	DD	DC
	n = 6	n = 6	n = 6	n = 6
Body weight (g) day 21	30±2	26±1	29±4	28±4
day 100	242±5	235±4	226±7	238±10
day 200	254±7	294±11	268±11	304±9*
Heart rate (bpm)	443±7	430±10	427±11	440±21
Systolic BP (mmHg)	106±4	96±3	93±6	108±5
11β-HSD1 (pg/ml)	19.1±7.7	2.0±1.2	28.3±7.3	22.4±14.1
Adiponectin (µg/ml)	7.06±0.31	7.46±0.73	7.83±0.34	7.19±0.79
Leptin (ng/ml)	4.42±0.55	4.77±0.67	4.31±0.32	3.74±0.39
Corticosterone (ng/ml)	795±45	796±78	861±28	789±70
Insulin (ng/ml)	1.69±0.13	2.05±0.21	1.99±0.20	2.61±0.11**
Glucose (mmol/L)	8.1±0.7	10.5±1.3	9.7±0.9	10.2±1.3
HOMA-IR	14.1±0.1	22.1±0. 3	19.8±0.2	27.3±0.1
HOMA-β (%)	170±25	135±32	148±20	180±29
iCa (mmol/L)	1.44±0.02	1.51±0.02	1.47±0.02	1.50±0.03

CC in which offspring of control-diet dams were nursed by control dams; DD in which offspring of Ca-deficient-diet dams were nursed by Ca-deficient-diet dams; CD in which offspring of control-diet dams were nursed by Ca-deficient-diet dams; and DC in which offspring of Ca-deficient-diet dams were nursed by control dams. No pups remained with their original birth mother; the pups in all groups came from different litters. BP: blood pressure

Statistically significant differences among the groups were determined with two-way analysis of variance (ANOVA) with diet and sex as the main factors. If interactions were found, the data were split, and a one-way ANOVA was performed. *P<0.05, **P<0.01 vs. CC.

Values are represented as means ± SE.

**Table 4:** Profile of male offspring on day 200.

	CC	CD	DD	DC
	n = 6	n = 6	n = 6	n = 6
Body weight (g) day 21	36±6	31±6	31±3	32±3
day 100	389±21	363±13	389±17	448±8*
day 200	418±7	419±15	430±12	472±18**
Heart rate (bpm)	400±15	401±12	394±10	384±12
Systolic BP (mmHg)	115±5	110±3	113±4	112±7
11β-HSD1 (pg/ml)	30.3±12.3	49.6±9.3	34.7±9.4	48.3±15.6
Adiponectin (µg/ml)	5.62±0.72	7.58±0.34*	7.30±0.45*	7.28±0.54*
Leptin (ng/ml)	6.36±0.72	6.90±0.47	7.38±0.45	7.93±0.52
Corticosterone (ng/ml)	580±26	556±24	628±18	615±23
Insulin (ng/ml)	2.21±0.10	2.46±0.20	3.02±0.20**	2.90±0.19*
Glucose (mmol/L)	9.1±0.7	9.9±0.6	12.6±1.8*	15.6±2.7**
HOMA-IR	20.6±0.1	25.0±0.1	39.1±0.4**	46.4±0.5**
HOMA-β (%)	182±26	178±20	153±22	111±26*
iCa (mmol/L)	1.39±0.02	1.33±0.03	1.37±0.03	1.38±0.04

CC in which offspring of control-diet dams were nursed by control dams; DD in which offspring of Ca-deficient-diet dams were nursed by Ca-deficient-diet dams; CD in which offspring of control-diet dams were nursed by Ca-deficient-diet dams; and DC in which offspring of Ca-deficient-diet dams were nursed by control dams. No pups remained with their original birth mother; the pups in all groups came from different litters. BP: blood pressure

Statistically significant differences among the groups were determined with two-way analysis of variance (ANOVA) with diet and sex as the main factors. If interactions were found, the data were split, and a one-way ANOVA was performed. *P<0.05, **P<0.01 vs. CC.

Values are represented as means ± SE.

### HOMA-IR and serum adipokine levels

In males, the mean levels of insulin, glucose, and HOMA-IR in DD and DC offspring were higher than those in CC pups (Table 4). Thus, male offspring from Ca-deficient dams developed insulin resistance. In females, the mean insulin levels in DC pups were higher than those in CC offspring (Table 3). In males, mean HOMA-β levels in DC offspring were lower than those in CC pups (Table 4). Thus, lactation from cross-fostered Ca-deficient dams affected insulin secretion in offspring. No significant difference was found among the groups in serum 11β-HSD1 levels (Tables 3 and 4).

In male offspring, mean adiponectin levels from CD, DD, and DC offspring were higher than levels in CC pups. No significant differences were found in serum ionized Ca, corticosterone, or leptin levels in offspring (Tables 3 and 4). Serum ionized Ca showed a pronounced sexual dimorphism, with higher levels in females (P<0.05).

### Maternal dietary calcium deficiency alters offspring mRNA expression of *Hsd11b1* in the liver

**Figure 1:**
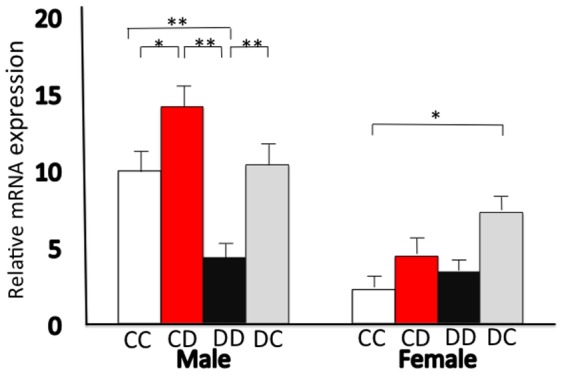
Hepatic mRNA expression levels of *Hsd11b1*. After delivery, foster mothers fed the same or different diets: CC, DD, CD, and DC (first letter: diet of original mother; second letter: diet of nursing mother). Data are represented as the means ± standard error. Statistically significant differences among the groups were determined with two-way analysis of variance (ANOVA), with diet and sex as the main factors. If interactions were found, the data were split and analyzed with one-way ANOVA. *P<0.05, **P<0.01.

We found a significant effect of sex (P<0.0001) on *Hsd11b1* mRNA expression, which was lower in females. *Hsd11b1* mRNA expression was significantly lower in male DD offspring than in the other male groups (Figure 1). In males, *Hsd11b1* mRNA expression in cross-fostered offspring was higher than that in offspring nursed by dams fed the same diet: in males, *Hsd11b1* mRNA expression in CD pups was higher than that in CC pups, and expression in DC offspring was higher than that in DD offspring. In addition, the *Hsd11b1* mRNA expression in female DC offspring was higher than that in CC pups (Figure 1).

## Discussion

This study shows that male offspring developed insulin resistance after nursing from Ca-deficient dams. Recently, epigenetic regulation has been shown to contribute to differences in fetal gene expression [31]. For example, we reported hypomethylation but decreased mRNA expression of hepatic *Hsd11b1* in offspring of dams fed a Ca-deficient diet during pregnancy [24]. Serum corticosterone levels of male pups from the Ca-deficient dams were higher than that from the control on day 21. These data indicate that maternal Ca deficiency in rats may have contributed to prenatal programming of insulin resistance. Previous reports have shown that the epigenetic effect induced by intrauterine growth restriction is gender specific [34], [35], [36]. Testosterone may affect epigenetic regulation; however, further investigation is necessary. Serum ionized Ca showed pronounced sexual dimorphism, with higher levels in females (P<0.05). 11β-HSD1 also shows pronounced sexual dimorphism in the liver, with lower levels in females [37]. In addition, the phenotype in males resulting from fostering is exaggerated compared with that of females, particularly in relation to adiposity and blood pressure [38]. It should be denied that the cross-fostering does not affect this result due to the role of non-maternal nutrition.

We previously reported that *Hsd11b1* expression in the liver increases in response to a Ca-deficient diet in adult rats for 2 wk [23]. In our current study, however, the expression of *Hsd11b1* mRNA in male offspring (DD) from Ca-deficient dams was lower than that in male offspring (CC) from control dams. Down-regulation of *Hsd11b1* suggests that a compensatory mechanism may diminish cortisol production in the liver. Reduced hepatic glucocorticoid exposure also represents a compensatory mechanism that limits the metabolic complications of insulin resistance. No significant difference was found among the groups in serum 11β-HSD1 levels; however, this may have been due to tissue-specific differences between serum and liver. Whether glucocorticoids modulate *Hsd11b1* expression is unknown; hepatic *Hsd11b1* expression is very different from that in other tissues [43], [44], [46]. Mice that overexpress *Hsd11b1* in adipose tissue develop visceral obesity and insulin resistance [39]. Obese rodents exhibit tissue-specific dysregulation of 11β-HSD1, usually with up-regulation in adipose tissue and down-regulation in liver [40], [41]. In both obese Zucker rats and obese humans, 11β-HSD1 activity is high in adipose tissue, but low in liver [42], [43], [44]. In adipose tissue and smooth muscle cells, glucocorticoid induces *Hsd11b1* mRNA expression, but controversial results have been obtained in the liver [42], [43]. Whether such tissue-specific dysregulation occurs in obese patients with type 2 diabetes is uncertain. Down-regulation of 11β-HSD1 in the liver in euglycemic obesity can be viewed as an adaptive process to decrease intra-hepatic glucocorticoid concentrations [45]; failure to decrease 11β-HSD1 in type 2 diabetes may result in increased intrahepatic cortisol levels, which exacerbates the adverse metabolic phenotype.

Our data show that metabolic programming effects are modified by nutrition in the immediate postnatal period. Hepatic *Hsd11b1* expression in cross-fostered male offspring (DC and CD) was higher than that in offspring (DD and CC) nursed by dams fed the same diet. Mismatched nutrition after birth, i.e., cross-fostered nursing, disrupts the adaptation that has been programmed in the fetus. Although hepatic expression of *Hsd11b1* in offspring from Ca-deficient dams may have been originally up-regulated by epigenetic mechanisms, *Hsd11b1* was likely down-regulated by other mechanisms during the early postnatal period. If the nutritional environment established before birth is mismatched after birth, the balance established by this compensation collapses. Metabolic programming effects translate into an adverse intrauterine environment during pregnancy. Altered mismatched nutritional experience during the suckling period can impact adult health in the offspring [47], [48]. These findings lead to the hypothesis that factors present in breast milk may regulate the epigenetics of pups. In addition, our data showed that cross-fostered lactation induced higher body weight. These early adaptations in the liver (expression levels of *Hsd11b1*) and hypothalamus (supporting hyperphagia and increased body weight gain) persist in the post-weaning period. Thus, nutrition during the prenatal period and/or lactation may affect epigenetic mechanisms in the offspring. Although the precise mechanisms that support metabolic programming effects due to altered nutritional experiences in the immediate postnatal period are not well understood, altered *Hsd11b1* mRNA expression has been implicated in this process [49].

We conclude that maternal Ca restriction during pregnancy alters postnatal growth, *Hsd11b1* expression, and insulin resistance in a sex-specific manner. The present study provides further support for the hypothesis that early postnatal lactation plays a sexually divergent role in programming the phenotype later in life.

## Supporting Information

Table S1 Vitamin mixture, supplied by CLEA Japan, Inc., Tokyo, Japan.
(DOCX)

Table S2 Calcium deficient mineral mixture, supplied by CLEA Japan, Inc., Tokyo, Japan.
(DOCX)
