# Supplementary material for: A Calcium-Deficient Diet in Rat Dams during Gestation and Nursing Affects Hepatic 11β-hydroxysteroid dehydrogenase-1 Expression in the Offspring
Source: PLoS One. 2014 Jan 10;9(1):e84125. doi: 10.1371/journal.pone.0084125 (PMC3888454; doi:10.1371/journal.pone.0084125)
Supplement: Table S1 — Vitamin mixture, supplied by CLEA Japan, Inc., Tokyo, Japan. (DOCX) [file pone.0084125.s001.docx]

1. Vitamin mixture, supplied by CLEA Japan, Inc., Tokyo, Japan.

| Ingredients | (mg/100g diet) |  |
| --- | --- | --- |
| Corn starch | 635.378 |  |
| Cholin chloride | 300.000 |  |
| Vitamin E (50%) | 20.000 |  |
| Inositol | 15.000 |  |
| PABA | 10.150 |  |
| Nicoticic acid | 10.150 |  |
| VitaminB_12_ (2%) | 0.250 |  |
| D-pantothenic acid | 2.000 | |
| Vitamin A (1,000,000IU/g) | 1.200 | |
| Vitamin D_3_(500,000IU/g) | 0.480 | |
| Vitamin B_2_ | 1.872 | |
| Vitamin B_1_ | 1.500 | |
| Vitamin B_6_ | 1.020 | |
| Biotin (2%) | 0.500 | |
| Vitamin K_3_ | 0.300 | |
| Folate | 0.200 | |
| Total | 1,000.000 | |
