# Supplementary material for: A Calcium-Deficient Diet in Rat Dams during Gestation and Nursing Affects Hepatic 11β-hydroxysteroid dehydrogenase-1 Expression in the Offspring
Source: PLoS One. 2014 Jan 10;9(1):e84125. doi: 10.1371/journal.pone.0084125 (PMC3888454; doi:10.1371/journal.pone.0084125)
Supplement: Table S2 — Calcium deficient mineral mixture, supplied by CLEA Japan, Inc., Tokyo, Japan. (DOCX) [file pone.0084125.s002.docx]

1. Calcium deficient mineral mixture, supplied by CLEA Japan, Inc., Tokyo, Japan.

| Ingredients | (mg/100g diet) |  |
| --- | --- | --- |
| Corn starch | 2,803.96 |  |
| KH_2_PO_4_ | 1,730.00 |  |
| NaH_2_PO_4_・2H_2_O | 1,361.71 |  |
| MgSO_4_・7H_2_O | 800.00 |  |
| FeC_6_H_5_O_7_・5H_2_O | 190.00 |  |
| NaCl | 89.73 |  |
| MnSO_4_・4H_2_O | 15.4 |  |
| 2ZnCO_3_・3Zn(OH) _2_・H_2_O | 6.00 | |
| Ca(IO_3_) _2_ | 1.54 | |
| CuSO_4_・5H_2_O | 1.26 | |
| CoCl_2_・6H_2_O | 0.40 | |
| Total | 7,000.00 | |
